# Surgical accuracy of coronal and sagittal alignment in conventional closed-wedge high tibial osteotomy after computer-assisted surgery experience

**DOI:** 10.1186/s43019-023-00205-2

**Published:** 2023-12-21

**Authors:** Sang Jun Song, Dae Kyung Bae, Se Hwan Park, Cheol Hee Park

**Affiliations:** 1 Department of Orthopaedic Surgery, Kyung Hee University College of Medicine, Kyung Hee University Medical Center, 26 Kyunghee-Daero, Dongdaemun-Gu, Seoul, 02447 Korea; 2 Department of Orthopaedic Surgery, Seoul Sacred Heart General Hospital, Seoul, Korea

**Keywords:** Knee, High tibial osteotomy, Computer-assisted surgery, Navigation

## Abstract

**Background:**

Although intraoperative navigation can improve the surgeon’s proficiency, no studies have analyzed postoperative outcomes of high tibial osteotomy (HTO) after computer-assisted surgery (CAS) experience. The present study compared the clinical and radiographic results between conventional and CAS closed-wedge (CW) HTOs after CAS experience.

**Methods:**

Each of the 50 conventional and CAS CW HTOs performed by single surgeon between 2015 and 2017 were included. The surgeon had experience of 140 cases of CAS CW HTOs before the study period. The groups were not different in terms of demographics. Clinically, the Western Ontario and McMaster Universities Osteoarthritis Index (WOMAC) and range of motion (ROM) were investigated. Radiographically, the mechanical axis (MA), change in posterior tibial slope angle (PTS), and parallel angle were evaluated. The proportions of inlier groups for the postoperative MA (within valgus 2° ± 3°), change in the PTS (within ± 3°), and parallel angle (< 3°) were compared.

**Results:**

There were no significant differences in postoperative clinical results between the conventional and CAS groups. The MA was appropriately corrected in both groups (2.4° versus 2.9°, *p* = 0.317). The amount of change in PTS was significantly greater in the conventional group (−2.2° versus −0.8°, *p* = 0.018). The parallel angle was 5.3° in the conventional groups and 3.1° in the CAS group (*p* = 0.003). The proportion of inlier group was not significantly different in the postoperative MA (72% versus 78%) and change in the PTS (52% versus 66%). The proportion of inlier for the parallel angle was significantly lower in the conventional group (36% versus 60%, *p* = 0.027).

**Conclusions:**

The surgical proficiency after CAS experience could cover the advantages of an intraoperative navigation in coronal adjustment, not in the sagittal adjustments in CW HTOs. A larger cohort with multiple surgeons in multiple centers would be required to identify the general trend.

**Study design:**

Level of evidence III.

## Introduction

Computer-assisted surgery (CAS) using intraoperative navigation allows the surgeon to check dynamic measurements of the limb alignment in three dimensions during high tibial osteotomy (HTO). The CAS can allow the surgeon to perform immediate intraoperative correction by providing real-time feedback. Many previous studies have reported accurate postoperative coronal and sagittal alignment in CAS HTOs [1]. The authors also reported the advantages of CAS compared to conventional surgery when performing closed-wedge (CW) HTO [2, 3].

Intraoperative navigation can improve the surgeon’s proficiency in the surgical procedure [4]. It implied that the results related with surgical accuracy of conventional HTOs can improve after CAS experience [5–7]. However, no studies have analyzed the postoperative outcomes in conventional HTO after CAS experience or have compared outcomes between conventional and CAS HTOs after CAS experience. A study addressing this issue would provide information to assess the degree to which the surgeon’s proficiency can overcome the benefit of CAS in terms of the surgical accuracy and evaluate the substantive usefulness of CAS in HTO.

The aim of this study was to compare the clinical and radiographic results between conventional and CAS CWHTOs performed by a single surgeon after CAS experience. It was hypothesized that the results of the conventional and CAS CWHTOs would be comparable.

## Patients and methods

### Patients

The conventional and CAS CW HTOs performed in our hospital between 2015 and 2017 were retrospectively searched. Intraoperative navigation was randomly used in the study period, although the surgeon tried to use it as much as possible when it was available for hire from the manufacturer. All procedures were performed by a single surgeon using the similar technique. The surgeon had experience with consecutive 140 cases of CAS CW HTOs prior to the study period (between 2009 and 2014) [2]. Surgical indication for CW HTO were cases with (1) medial compartment osteoarthritis (Kellgren-Lawrence Grade 2–3) associated with a varus deformity, (2) flexion contracture < 10°, (3) flexion angle > 90°, and (4) without lateral compartment osteoarthritis and lateral tibial subluxation of > 1 cm.

The inclusion criteria were patients with (1) a minimum postoperative follow-up period of 5 year and (2) available appropriate medical records and preoperative and postoperative radiographs. The exclusion criteria were as follows: (1) history of trauma, infection, or previous surgery on either knee or (2) instability of either knee owing to any ligament problem.

According to the criteria, initially, 62 conventional CW HTOs and 50 CAS CW HTOs performed between 2015 and 2017 were identified. The 50 conventional CWHTOs were selected and matched to the 50 CAS CW HTOs according to age, sex, body mass index, preoperative mechanical axis (MA), and posterior tibial slope (PTS). There was no significant difference in the demographics and preoperative knee conditions between the conventional and CAS groups (Tables 1, 2, 3). Informed consent was obtained from all patients before the review. This study was approved by the institutional review board of our hospital.

**Table 1:** Demographics

	Conventional	CAS	*p* value
Number of knees	50	50	
Age (year)	58.5 ± 4.4	56.9 ± 6.5	0.147
Female/male	42/8	41/9	1.000
Body mass index (kg/m^2^)	25.9 ± 3.4	25.7 ± 3.3	0.790

**Table 2:** Clinical results

		Conventional	CAS	*p* value
WOMAC	Preoperative	69.7 ± 5.7	69.9 ± 5.8	0.904
	Postoperative	18.1 ± 2.8	17.5 ± 2.5	0.307
Range of motion (°)	Preoperative	132.6 ± 7.6	132.2 ± 10.6	0.837
	Postoperative	135.6 ± 7.4	135.2 ± 14.4	0.862

Data are presented as mean ± standard deviation

*CAS*  computer assisted surgery, *WOMAC*  Western Ontario and McMaster universities Osteoarthritis Index

**Table 3:** Radiographic results

		Conventional	CAS	*P* value
Mechanical axis (°)	Preoperative	Varus 7.6 ± 3.5	Varus 8.2 ± 2.1	0.307
	Postoperative	Valgus 2.4 ± 2.7	Valgus 2.9 ± 2.6	0.317
Medial proximal tibial angle (°)	Preoperative	84.0 ± 2.7	83.7 ± 3.1	0.624
	Postoperative	93.1 ± 2.8	93.4 ± 2.0	0.605
Posterior tibial slope angle (°)	Preoperative	11.2 ± 3.3	10.5 ± 3.3	0.267
	Postoperative	9.0 ± 3.3	9.7 ± 3.2	0.283
	Change	− 2.2 ± 3.1	− 0.8 ± 2.9	0.018
Parallel angle (°)		5.3 ± 4.4	3.1 ± 2.8	0.003

Data are presented as mean ± standard deviation

*CAS*  computer-assisted surgery

### Surgical technique and rehabilitation

Conventional and CAS CW HTOs were performed using the similar surgical technique, with the exception of the intraoperative navigation system and the number of guide pins (two or four pins) used on the proximal and distal osteotomy surfaces [3]. The postoperative MA were targeted to be valgus 2° [8, 9]. In conventional HTO, one pin was inserted in the proximal plane of the osteotomy and the other in the distal plane under fluoroscopic guidance to determine the correction and wedge size on preoperative radiographic planning. The alignment was confirmed with a rod. In CAS HTO, the VectorVision CT-free navigation system (Ver 1.1; Brainlab, Heimstetten, Germany) was used. A precalibrated navigation drill guide was used to place each two pins in the proximal and distal planes of the osteotomy. The anterior and posterior heights of the wedge were rendered equivalent using the four-pin technique. A miniplate staple (U&I, Uijeongbu-si, Korea) was used to fix the osteotomy site. A similar rehabilitation protocol was used for all patients; isometric exercises were recommended on the operative day, straight-leg raising exercises were started one day postoperatively, partial weight-bearing was begun 4 days postoperatively, and full weight bearing without crutches was started at 4–8 weeks on the basis of the patient’s condition.

### Clinical evaluation

The clinical results were evaluated preoperatively and at 5 years postoperatively according to the Western Ontario and McMaster Universities Osteoarthritis Index (WOMAC) and range of motion (ROM).

### Radiographic evaluation

**Fig. 1:**
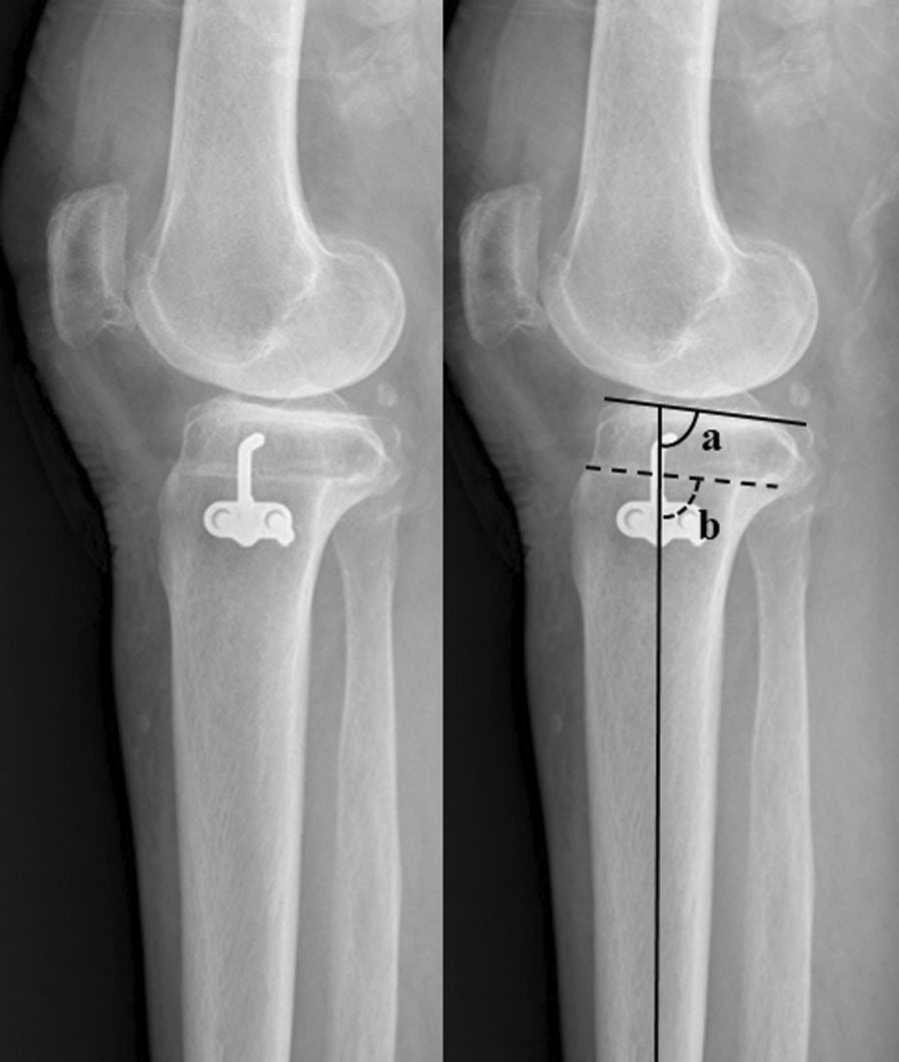
The parallel angle. The parallel angle was defined as the absolute value of the angle between the medial joint surface of the and the osteotomy plane (|a–b|)

The radiographic parameters were measured on preoperative radiographs and radiographs taken 3 months postoperatively taken under weight bearing. Measurements of the MA and medial proximal tibial angle (MPTA) were taken from orthoroentgenograms showing the hip, knee, and ankle. The MA was defined as the angle between the femoral and tibial mechanical axes [10]. The MPTA was the medial angle between two lines (i.e., the line of the tibial mechanical axis and the tangent line of the tibial plateau) [11]. Lateral radiographs of the knee were used to measure the PTS and parallel angle. The PTS was measured as the angle formed by a line perpendicular to the reference line and medial tibial plateau [10]. The tibial intramedullary reference line of the PTS was defined as the line connecting the center of the medullary canal at 10 and 20 cm distal to the tibial plateau. The change in the PTS was defined as the postoperative minus preoperative PTS, with a larger value indicating a slope increase. The parallel angle was defined as the absolute value of the angle between the medial joint surface and the osteotomy plane, with a larger value indicating a steeper osteotomy line to the medial joint surface (Fig. 1) [2].

The quality of radiographic evaluations was improved by the radiographic protocol of standardizing the position of the knee and thus creating an identical distance between the X-ray beam and cassette for each patient [10]. The images were transferred digitally to a picture archiving and communication system (PACS; Infinitt, Seoul, Korea) and were then assessed. The minimum difference that the software could detect was 0.1° in angle. Two independent investigators carried out the radiographic measurements to reduce observation bias. The interobserver reliabilities of the measurements were assessed using an intraclass correlation coefficient. The intraclass correlation coefficients for all measurements were greater than 0.8. Thus, the average values of the two investigators were used.

### Incidence of failure

An incidence of failure was investigated during 5 years of follow-up; the failure was defined as conversion to total knee arthroplasty. Additionally, any complications that may have occurred, including implant breakage, pin site fracture, or infection, were also investigated.

### Statistical analysis

The clinical results (WOMAC, and ROM) and radiographic results (MA, MPTA, PTS, change in the PTS, and parallel angle) were compared using independent *t*-test. The proportions of inlier groups for the postoperative MA (within valgus 2° ± 3°), change in the PTS (within ± 3°), and parallel angle (< 3°) were compared by the chi-squared test [2, 12, 13]. Statistical analyses were performed using SPSS version 25.0 (SPSS Inc., Chicago, IL, USA), and a *p*-value < 0.05 was considered statistically significant.

Post hoc power analyses with an alpha of 0.05 were performed to check whether the sample had sufficient power to detect significant differences. A power > 80% was considered sufficient, and all of the variables that were significantly different met this criterion.

## Results

Clinically, there were no significant differences in terms of WOMAC and ROM between the two groups pre- and postoperatively (Table 2). Radiographically, the MA and MPTA were appropriately corrected in both groups (Table 3). The average PTS decreased in both group; the amount of change in PTS was significantly greater in the conventional group (−2.2° versus −0.8°, *p* = 0.018). The parallel angle was 5.3° in the conventional group and 3.1° in the CAS group (*p* = 0.003).

**Fig. 2:**
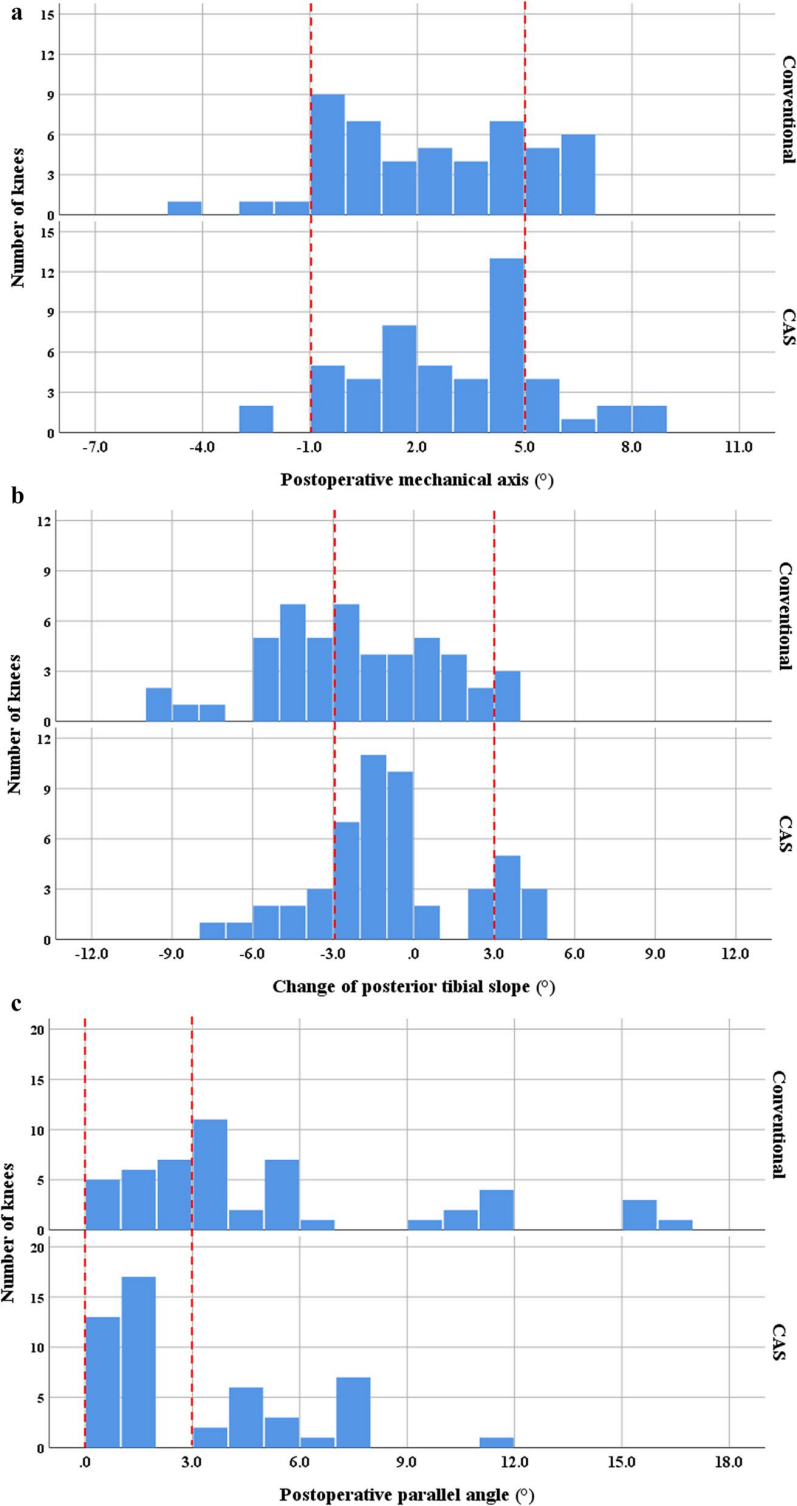
The percentages of inliers for the postoperative mechanical axis (MA) (within valgus 2° ± 3°), change in posterior slope (PTS) (within ± 3°), and the parallel angle (< 3°). **A** The percentages of appropriate postoperative MAs were 72% and 78% in the conventional and CAS groups (*p* = 0.645). **B** The percentages of well-preserved PTS were 52%, and 66% in the conventional and CAS groups (*p* = 0.222). **C** The percentages of appropriate parallel angles were 36%, and 60% in the conventional and CAS groups (*p* = 0.016)

The proportion of inlier group was not significantly different in the postoperative MA and change in the PTS (Fig. 2A, B). The proportion of inlier for the parallel angle was significantly lower in the conventional group (36% vesus 60%, *p* = 0.027) (Fig. 2C).

No failure was observed in both groups during 5 years of follow-up. Furthermore, complications were not observed during the follow-up.

## Discussion

The most important finding was that there were no significant differences in coronal adjustments, but not in sagittal adjustments between the conventional and CAS CW HTOs, after sufficient CAS experience.

An intraoperative navigation helps the surgeon to achieve an accurate position for the hinge axis, which affects postoperative coronal and sagittal alignment, as well as symmetric wedge resection associated with the change in the PTS during CW HTO [2, 12]. Additionally, the surgeon can evaluate the change in the postoperative MA response to an external varus or valgus force when using navigation; such evaluation provides confirmation that there is no cortical hinge fracture, as well as information regarding postoperative fixation stability and changes in limb alignment during weight bearing [14].

These navigation features appear to enable surgeons to develop and refine their technique and judgment in CW HTO procedures. Navigation provides useful information on how to perform the delicate phase of the procedure better, with greater attention to detail. It allows for verification of the precision of each individual surgical step and provides the ability to verify limb alignment. There has been considerable inherent agreement that intraoperative navigation is a valuable tool for improving surgical skills [4–7]. Love et al. [6] demonstrated that navigation has training benefits in terms of increasing awareness of surgical errors. Furthermore, Iorio et al. [5] revealed that the use of intraoperative navigation can improve surgical accuracy, even among experienced and high volume arthroplasty surgeons.

In the present study, the surgeon had experience with 140 consecutive cases of CAS CW HTOs prior to the study period, which would be sufficient to improve the surgical proficiency. After CAS experience, there were comparable results related with coronal alignment between the conventional and CAS CWHTOs. The surgical proficiency after CAS experience seems to be able to cover the benefit of CAS in terms of coronal adjustments in CWHTOs.

Yan et al. [1] reported that navigation preserved the PTS significantly better than did conventional HTO, but their systematic review did not demonstrate a significant difference in the achievement of an appropriate MA. Considering this study, it would be inferred that sagittal adjustment is more difficult than coronal alignment adjustment during HTO. In our study, average amount of change in PTS was significantly greater in conventional CW HTOs even with improved surgical proficiency after CAS experience. Regarding the parallel angle, representing the orientation of the wedge, which is related to the change of PTS, the average value and the proportion of inlier were more appropriate in the CAS group [2]. An intraoperative navigation will be helpful in more precise adjustment of sagittal alignment during CW HTOs, even for surgeons with improved sufficient proficiency.

While navigation has an advantage in sagittal alignment adjustment, it may require a considerable amount of CAS experience for this advantage to become more noticeable. Song et al. [15] reported that the accuracy and precision of postoperative coronal alignment improved after the first 70 cases when performing CAS CW HTOS. However, no study has analyzed the number of cases required to overcome the learning curve for sagittal alignment adjustment in CAS CW HTOs. Further research is necessary to address this issue in CAS CW HTOs.

Most previous studies reported no significant differences between conventional and CAS HTOs in terms of clinical results despite showing significant differences in the radiographic results [16–19]. A systematic review showed that the increase in the Lysholm score was 50.8 for conventional HTO and 52.1 for CAS HTO, with no significant difference (*p* = 0.136) [1]. Such nonsignificant clinical differences were consistent with our results. This might be because the validated clinical rating systems may not be sensitive enough to detect subtle changes. This ceiling effect has to be considered when comparing the clinical outcomes of the conventional and CAS HTOs.

Previous studies, suggesting the benefit of CAS, have compared the surgical accuracies of the conventional HTOs before CAS experience and CAS HTOs [2, 3]. Because the surgical proficiency can improve over time even without CAS, comparative studies of such a design could not clearly show pure benefits of CAS. In this context, the present study is valuable in showing the pure benefit of CAS for sagittal alignment by comparing the results between the conventional HTOs after CAS experience and CAS HTOs.

This study had several limitations. First, the study has a retrospective design with a possibility of selection bias. However, matched pairing was performed to make up for this inherent limitation. Second, the sample size was small. Although there was no statistical significance, the proportion of inlier of postoperative coronal alignment tended to be more precise in CAS CWHTOs. If the sample size was larger, the interpretation of our results may have changed. However, it can be valuable to present the significant differences in sagittal adjustments even with a small sample size. Third, the HTO procedure in this study was performed by a single surgeon. Caution must be taken when extrapolating these findings to different countries and hospitals.

In conclusion, the surgical proficiency after CAS experience could cover the advantages of an intraoperative navigation in coronal adjustment, not in the sagittal adjustments in CWHTOs. A larger cohort with multiple surgeons in multiple centers would be required to identify the general trend.

## Data Availability

The datasets generated and/or analyzed during the current study are not publicly available, but they are available from the corresponding author on reasonable request.
